# The Role of Phytosterols in Nonalcoholic Fatty Liver Disease

**DOI:** 10.3390/nu14112187

**Published:** 2022-05-24

**Authors:** Otilia Frasinariu, Roxana Serban, Laura Mihaela Trandafir, Ingrith Miron, Magdalena Starcea, Ioana Vasiliu, Anna Alisi, Oana Raluca Temneanu

**Affiliations:** 1 Department of Mother and Child, “Grigore T. Popa” University of Medicine and Pharmacy, 700115 Iasi, Romania; frasinariu.otilia@umfiasi.ro (O.F.); ingrithmiron@hotmail.com (I.M.); magdabirm@yahoo.com (M.S.); ralucatemneanu@yahoo.com (O.R.T.); 2 Department of Biochemistry, “Grigore T. Popa” University of Medicine and Pharmacy, 700115 Iasi, Romania; 3 Department of Physiology, “Grigore T. Popa” University of Medicine and Pharmacy, 700115 Iasi, Romania; ioana_medgen@yahoo.com; 4 Research Unit of Molecular Genetics of Complex Phenotypes, Bambino Gesù Children’s Hospital, IRCCS, 00165 Rome, Italy; annaalisi@opbg.net

**Keywords:** NAFLD, phytosterols, stigmasterol, β-sitosterol

## Abstract

Nonalcoholic fatty liver disease is now recognized as the most common cause of chronic liver disease with an increasing prevalence in both adults and children. Although the symptoms are absent or poorly expressed in most cases, some patients may progress to end-stage liver disease. The pathogenesis of NAFLD is known to be multifactorial. Current therapeutic recommendations focus on lifestyle changes in order to reduce the incidence of risk factors and drugs targeting major molecular pathways potentially involved in the development of this disease. Given that a pharmacological treatment, completely safe and effective, is not currently known in recent years more research has been done on the effects that some bio-active natural compounds, derived from plants, have in preventing the onset and progression of NAFLD. Numerous studies, in animals and humans, have shown that phytosterols (PSs) play an important role in this pathology. Phytosterols are natural products that are found naturally in plant. More than 250 phytosterols have been identified, but the most common in the diet are stigmasterol, β-sitosterol, and campesterol. Consumption of dietary PSs can reduce serum cholesterol levels. Due to these properties, most studies have focused on their action on lipid metabolism and the evolution of NAFLD. PSs may reduce steatosis, cytotoxicity oxidative stress, inflammation, and apoptosis. The purpose of this review is to provide an overview of the importance of dietary phytosterols, which are a window of opportunity in the therapeutic management of NAFLD.

## 1. Introduction

Nonalcoholic fatty liver disease (NAFLD) is the most common cause of chronic liver disease worldwide, and it is probably to become the leading cause of end-stage liver disease in the next decades [1]. The disease encompasses a broad spectrum of liver damage, from steatosis, that refers to more than 5% ectopic fat accumulation in the liver, to steatohepatitis, and leads to more severe complications including fibrosis, cirrhosis, and even hepatocellular carcinoma [2]. Over the years, NAFLD was indicated as the hepatic manifestation of metabolic syndrome. Recently, an international panel of experts have proposed the change of the terminology of this disease, right now NAFLD being defined like metabolic-associated fatty liver disease (MAFLD) [3,4]. This new terminology intents to comprise the strict connection between hepatic steatosis and metabolic disorders such as T2DM, obesity, dyslipidemia, and hypertension, which increase the risk of disease progression and development of NASH and fibrosis [5]. 

NAFLD became a public health issue due to the fact that almost 25% of the world’s population is affected [6]. The increased rates of obesity and overweight in the last decades has led to the increase in the worldwide prevalence of NAFLD, especially in obese patients in which the prevalence is up to 75% [7]. A worrying phenomenon is the increase of NAFLD prevalence among children, estimated to be around 7.6%, rising up to 50–70% among obese children [8]. 

Initially, the pathogenesis of NAFLD started from the “two-hit theory” [9]. Based on current knowledge, the widely accepted theory is the “multifactorial theory” [10]. “Multiple-hit” hypothesis states that the pathogenesis of NAFLD results from a combination of genetic predisposition and environmental factors, like high-fat diet, sedentary lifestyle, sleep deprivation, along with lipotoxicity, altered gut-microbiome, cytokines, and adipocytokines [11,12]. The first step in the disease is represented by the fat accumulation in the liver [10]. Simultaneously, there is a complex network of interrelations between various organs, the initial stage of NAFLD development not being limited to liver involvement. The second hits in NAFLD includes the effect of inflammatory cytokines, adipokines, mitochondrial destruction, as well as changes in the gut microbiota and promotes the effect of oxidative stress, all this leading to steatohepatitis and fibrosis [11,12]. Among the many parallel strokes, oxidative stress is the main factor contributing to the development of liver damage and disease progression. Both exogenous factors—viruses, alcohol, an unbalanced diet, drugs-, and endogenous—such as insulin resistance, obesity, and diabetes—may promote oxidative stress in liver disease [13]. These parallel hits have been studied as potential therapeutic targets of NAFLD [14,15,16]. 

Due to its complex pathogenesis, there is currently no cure for the disease itself. There is an urgent need to identify novel therapeutic interventions to reduced lipotoxicity and hepatic inflammation. NAFLD therapy should aim at removing fat accumulations from hepatocytes and prevent or correct lipotoxicity-induced liver damage [2,16]. Given the multi-factorial pathogenesis of the disease, current therapeutic approaches consist of strategies to reduce the incidence of risk factors through lifestyle changes (e.g., obesity, dyslipidemia, insulin resistance) and drugs targeting major molecular pathways potentially involved in the development of this disease [17]. In the first plan of actions are the lifestyle changes, diet and exercise, weight loss and insulin-sensitizing agents, which are meant to correct insulin resistance, hyperinsulinemia, and reduce fat mass, in particular, visceral adiposity [18]. The second strategy in NAFLD treatment is to prevent or to reverse hepatocellular damage by inhibiting lipid peroxidation and oxidative stress or by using anti-inflammatory, anti-apoptotic, or other hepatoprotective agents [2,16,19]. The pharmaceutical therapies available for clinical treatments are limited due to the complex pathogenesis and variate types of lesions. The current pharmacological treatment options for NAFLD are insulin-sensitizers such as metformin, ursodeoxycholic, and statins [18,19]. However, some are expensive or had some adverse effects. Any proposed drugs have not provided solid results. 

Thus, the strategies for approaching the prevention and treatment of NAFLD are now focused on the role of dietary supplements, nutritional formulas, and development of new drugs [20]. Current studies indicate that the best results are achieved through a personalized multidisciplinary approach [21]. Lifestyle interventions, like dietary changes and exercise, should be combined with the administration of so-called bio-active natural compounds, such as polyphenols, polyunsaturated fatty acids, vitamin E, quercetin, phytosterols, fructo-oligosaccharides, and carbohydrate-dietary fibers [2,22]. Numerous clinical studies confirmed the ability of some of this natural product metabolites to exert beneficial effect(s) in the cellular mechanisms involved in the onset and progression of NAFLD [20,23]. Moreover, several studies published in the literature in recent years have confirmed the beneficial effects of phytosterols (PSs) on lipid metabolism, as serum cholesterol-lowering, and the evolution of NAFLD [24]. 

Due to the fact that phytosterols are less studied than other natural compounds in the NAFLD interventions, in this review, we summarize the most significant studies on animals and humans on the PSs role in NAFLD. 

## 2. Phytosterols: Composition, Occurrence, and Biological Activities

Phytosterols are natural products that are found in plants, in free or esterified forms. Phytosterols are classified into two groups: sterols—that are unsaturated compounds, and stanols—saturated molecules. In human diets and in plants, sterols are the main products, stanols representing only 10% of total intake of PSs [25]. 

### 2.1. Chemical Structures

Chemically, the structure of phytosterols is similar to that of cholesterol (C_27_H_46_O, cholest-5-en-3β-ol). The difference is that at C_24_ of PS is binding a metil (-CH_3_) group or etil (-CH_2_CH_3_) group. Thus, the new compound will have 28 or 29 carbon atoms [26].

**Figure 1:**
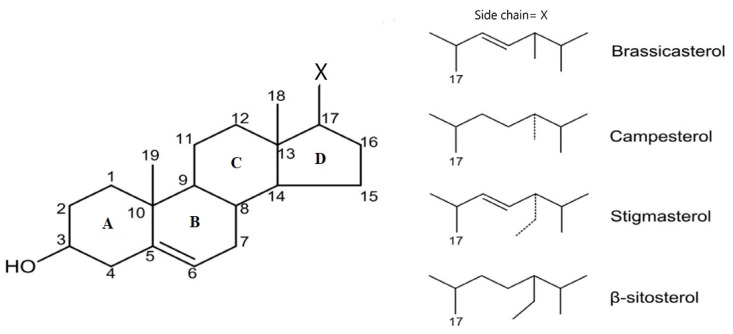
Main PSs chemical structure according to C-17 side chain.

Studies have identified more than 250 phytosterols. Phytosterols are formed by a tetracyclic cyclopenta (α) phenanthrene ring and an aliphatic C_17_ atom side chain. The main structures are represented by different variations of the lateral chain that bind to C_17_: brassicasterol, campesterol, stigmasterol, and β-sitosterol (Figure 1) [26,27].

Brassicasterol (C_28_H_46_O, 24-methyl cholest-5,22-dien-3β-ol) is a 3beta-sterol containing the hydroxyl group (OH) at C_3_ at the first sterol ring (A), elimination of C_24_.2 of the alkyl substituent. Furthermore, in ring B from carbon skeleton is presented an unsaturated bond in position C_5_-C_6_ and in position C_22_-C_23_ in the alkyl substituent [28]. 

Campesterol (C_28_H_48_O, cholest-5-en-24α-methyl-3β-ol) has the simplest chemical structure among sterols. It contains the OH group at C_3_ of the steroid skeleton and only one unsaturated bond at C_5_-C_6_ from the B ring [29].

Stigmasterol (C_29_H_48_O, stigmasta-5,22-dien-3-ol) is characterized by the OH group at C_3_ of the first steroid ring and unsaturated bonds between C_5_ and C_6_ position of the B ring and the C_22_-C_23_ position in the alkyl substituent. Stigmasterol is soluble in benzene, ether, ethanol, organic solvents, and water [30]. 

β-Sitosterol (C_29_H_50_O, cholest-5-en-24α-ethyl-3β-ol) chemical structure is similar to cholesterol: OH group at C_3_, double bond between C_5_ and C_6_. It presents an ethyl group at C_24_ [31].

### 2.2. Sources

PSs are found in plant foods and can be extracted from all parts of the plant. Phytosterols are present especially in whole grains, vegetables, fruits, legumes, nuts, seeds, and vegetable oils. Nuts and vegetable oils contain higher amount of PSs than cereals, vegetables, and fruits. β-Sitosterol, campesterol, and stigmasterol are the most common PSs in human daily diet [27]. They are found in large quantities in plants consumed by humans, the percentage in PSs total intake in diet being 65%, 30%, and 3%, respectively, but many of their properties are lost through heat processing [25]. The absorption depends on the solubility and the mixture from the food. We summarize the content in PSs in most common nuts and vegetal oils used in human diet (Table 1). PSs cannot be synthesized by humans. Various studies have found that an intake of about 2 g of phytosterols daily in the diet is ideal for reaping the many benefits of these substances [32].

**Table 1:** The main products that contains phytosterols [33].

**Product**	**Weight**		**Phytosterol Content**
Sesame oil	14 g (1 spoon)		118 mg
Sunflower oil	14 g (1 spoon)		60 mg
Olive oil	14 g (1 spoon)		30 mg
Dried soybean seeds	100 g		300 mg
Pumpkin seeds	100 g		94–265 mg
Sesame seeds	100 g		400 mg
Sunflower seeds	100 g		176–322 mg
Flaxseed	100 g		197–214 mg
Raw peas	75 g		133 mg
Pistachio	100 g		279–297 mg
Cashew	100 g		80–158 mg
Nuts	100 g		63–206 mg
Almond	100 g		89–208 mg
**PSs in oils as percentage of total sterol fraction**			
Product	β-Sitosterol	Campesterol	Stigmasterol
Sunflower oil	56–63	7–13	8–11
Olive oil	75.6–90	2.3–3.6	0.6–2
Coconut oil	50–70	7–10	12–18
Corn oil	55–67	7.2–8.4	1.2–1.8
Peanut oil	48–65	12–20	5–13
Soy bean oil	52–58	16–24	16–19

### 2.3. Biological Properties of PSs

PSs have been shown to have antioxidant, antipyretic, anti-inflammatory, and hormone-like functions, as well as anticancer, antidiabetic, antiatherosclerotic, and neuroactive effects [27]. PSs reduce total cholesterol and low-density lipoprotein cholesterol from plasma [34].

PSs are 5% absorbed in the small intestine through intestinal epithelial cells. PSs competes with cholesterol to form the micelles which are small aggregates containing lipids and bile acids. After esterification with acyl coenzyme A, PSs are incorporated into chylomicron. Unesterified PSs are eliminated into the intestinal content by way of ATP binding cassette (ABC). Once the chylomicron reaches the circulatory system through the lymph, it transfers free fatty acids to the peripheral tissues under the action of lipoprotein lipase and thus the remnants chylomicrons are formed which are taken up by the liver [35]. 

In the liver, PSs are metabolized to cholesterol and other products under the action of several enzymes. The most important enzyme is 7∝-hydroxylase cholesterol, which is the rate-limiting enzyme in bile acid biosynthesis reactions. Its actions are under a feedback control: it is inhibited by bile acids and stimulated by the amount of cholesterol. Bile acids are quickly discharged into bile by hepatic transporters ABC G5/G8. The rate of phytosterol secretion in the bile is higher than cholesterol secretion. Thus, low serum phytosterol levels can be explained by two mechanisms: increased PSs excretion in the bile and decreased intestinal absorption [25].

A percentage of 70–80% of PSs circulates in LDL particles. PSs can combine with the ABCA1 transporter in the basolateral membrane of intestinal cells and can enter the HDL composition. After being transported to the liver, PSs can return to the intestine via ABC from the hepatobiliary interface [26]. There are those who have shown that the administration of PSs has led to a decrease in total cholesterol and LDL cholesterol by decreasing the absorption of cholesterol from the diet and the action on the intestinal and hepatic mechanisms [36].

Consumption of PSs decreases liver inflammation. Desmosterol (C_27_H_44_O) along with endothelial nitric oxide synthase is a major regulator of inflammation with a role in maintaining mitochondrial function in the liver. The decline in nitric oxide synthase activity has been shown to be correlated with the progression of NAFLD [37,38]. Elevated desmosterol levels have been associated with liver inflammation. There is also an effect of PSs on immunity by inducing an activation in regulatory T-cells, by stimulating Th1 activity in mononuclear blood cells. The effect has been shown in patients with asthma, inflammatory bowel disease, and multiple sclerosis [24].

PSs decreases serum triacylglycerol (TAG) levels. This is done by: increasing lipoprotein lipase activity, increasing TAG uptake, influencing cholesteryl ester transfer protein (CETP) activity, decreasing intestinal lipid uptake or decreasing hepatic VLDL production [24].

## 3. The Role of Phytosterols in NAFLD 

**Figure 2:**
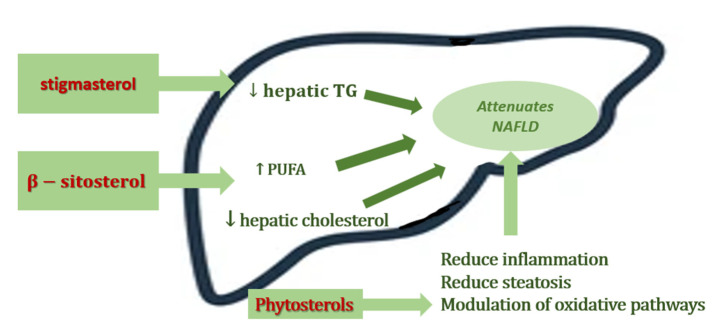
Beneficial effects of PSs in NAFLD.

The outlined above metabolic proprieties of phytosterols were the starting points for studying the potentially beneficial role in NAFLD prevention and management. These biologically active molecules have proven beneficial effects on lipid metabolism and in the prevention and progression of NAFLD, which we summarize in the Figure 2 [24]. Additional studies are needed to determine the individual impact of each compound [27]. 

We performed a PubMed and Medline database search using a combination of keywords, including NAFLD, NASH, phytosterols, sitosterol, campesterol, plant esters, and natural compounds, especially in vivo and in vitro studies. The articles were selected based on their relevance to the review; we included studies on animal models and humans with NAFLD, where the subjects involved were given PSs supplements. The main results are synthetized in Table 2.

**Table 2:** Studies on the effects of phytosterols in NAFLD.

Animal Models Studies					
	Study Design	Dietary Phytosterols Supplementation	Intervention	Biological Activity of Phytosterol	Reference
1	10–12 weeks old female low-density lipoprotein (LDL) receptor deficient mice, high-fat diet (HFD)	Plant sterol and stanols	Plant sterol esters (2%) or plant stanol esters (2%)	Lowered hepatic inflammation ↓ serum and hepatic cholesterol ↓ serum TAG concentrations	Plat J et al., 2014 [39]
2	Male apolipoprotein-E knockout mice, high fat diet	Flaxseed oil combined with its ester of plant sterols-treated (FO-PS)	3.3% (*w*/*w*) flaxseed oil ester of plant sterols mixture with amount of flaxseed oil	Improving hepatic steatosis ↓ ROS production ↓ inflammatory markers (IL-6, TNF, MCP-1, and ICAM-1	Han H et al., 2014 [40]
3	Five-week-old male Sprague Dawley rats, high-fat diet	Phytosterols esters (PSEs)	low and medium doses of PSEs (equal to 3 or 6 g/d in humans)	↓ LDL-C serum level; ↓ ALT, AST serum level; Modulating key cytokines; improved oxidative status ameliorate hepatic lesions	Song L et al., 2017 [41]
4	Adult Sprague Dawley rats, high-fat diet	Phytosterol esters (PSEs)	low-dose PSE (0.05 g per 100 g body weight) and high-dose PSE (0.10 g per 100 g body weight),	high-dose PSE treatment: - effectively inhibited the increase in liver and abdominal fat indexes and hepatic lipids - increased the relative abundance of Bacteroidetes and Anaerostipes	Song L et al., 2020 [42]
5	8-week old male C57BL/6 mice, high-fat modified Western-style diet	Stigmasterol β-sitosterol	0.4% stigmasterol or 0.4% β-sitosterol (comparable to that suggested for humans by the FDA)	↓ body weight gain ↓ serum cholesterol ↓ histological score ↓ ALT levels ↑ hepatic HMGCoAR mRNA expression ↓ the expression of lipogenic genes, FAS and SCD1 ↓ circulating CM levels	Feng S et al., 2018 [43]
6	78 week old male C57BL/6 mice, high-fat modified Western-style diet	Stigmasterol β-sitosterol	0.4% stigmasterol or 0.4% β-sitosterol	↓ triacylglycerols with polyunsaturated fatty acids decreased of free hepatic fatty acids increased MUFA and PUFA levels	Feng S et al. 2018 [44]
7	Growing male Sprague Dawley rats, high-fructose diet	β- sitosterol	+ 20 mg/kg β-sitosterol in a gelatine cube	↓ liver lipids ↓in liver mass ↓ areas of inflammation lesions of micro- and macrovesicular hepatic steatosis	Gumede NM et al., 2020 [45]
8	21-day-old female Sprague Dawley rat pups, high-fructose diet	β- sitosterol	+ 20 mg/kg β-sitosterol in a gelatine cube	↓ hypertriglyceridemia ↑ plasma adiponectin concentration ↓ plasma insulin concentration prevented the high-fructose diet-induced visceral obesity	Gumede NM et al., 2020 [46]
9	C57BL/6J mice, high fat and high cholesterol diet	Plant sterol ester of α-linolenic acid (PS-ALA)	3.3% PS-ALA	suppressed hepatic steatosis ↓ mitochondrial damage Inhibited Srebp Pathway Activation ↓ fatty acid accumulation ↓ oxidative stress ↓ lipid disorder ↓ release of IL-1β and IL-18 ↓ ALT, AST	Han H et al., 2019 [47]
**Clinical Trials**					
	**Study Design**	**Dietary Phytosterols Supplementation**	**Intervention**	**Results**	**Reference**
1	double-blind clinical trial 38 patients with NAFLD	phytosterols	1.6 g phytosterol supplementation daily	↓ LDL-C, TNF-α, ALT, AST	Javanmardi MA et al., 2018 [48]
2	cross-over clinical trial, 40 NAFLD subjects	phytosterols	1.8 g PSs supplementation daily for 4 weeks	↓ LDL-C ↓ systemic inflammation Improved insulin resistance	Chen et al., 2015 [49]
3	double-blind placebo-controlled trial 96 NAFLD subjects, overweight or dyslipidaemic	phytosterol esters + n-3 fatty acids	PS-enriched soy milk powder containing 3·3 g of PS plus fish oil capsules containing highly concentrated EPA and DHA (450 mg EPA + 1500 mg DHA), 12 weeks	↑ the liver: spleen attenuation ratio ↓ TAG levels ↓ TGF-β levels ↓ TNF-α levels ameliorate hepatic lesions	Song L et al., 2020 [50]

### 3.1. Animal Models’ Studies

PSs were investigated for diminishing inflammation through modulation of the immune cell’s inflammatory activity [51]. One of the first studies about the role of PSs in liver inflammation associated in NASH was performed by Plat et al. in 2014 [39]. Their study started from in vitro cells, continuing with mice and human analysis, and the studying hypothesis was that plant sterol/stanol esters reduces hepatic inflammation. The in vitro study showed that isolated bone-marrow derived macrophages incubated with LPS and sitostanol produced less Tnf-α, concluding that plant stanols could directly affect macrophages inflammation, independent of lipid metabolism [39]. In the animal model, supplementation of a high-fat diet (HFD) with plant sterol and stanol esters showed reduced inflammation in mouse liver, with less infiltrating macrophages (Mac-1) and neutrophils (NIMP) on immunohistochemical staining. The study also analyzed pro-inflammatory markers genes expression of the Cd68, Mcp-1, IL-1β, Tnf-α, ICAM in the hepatic tissue. These markers were significantly lowered in HFD enriched with plant sterol or stanol ester compared to the HFD alone. Moreover, synergic administration of plant sterols and HFD in mice showed a reduction of the serum and hepatic cholesterol precursors lathosterol/desmosterol levels compared with HFD fed mice [39].

Han H et al., 2014, studied the effects of flaxseed oil containing flaxseed oil ester of plant sterols on hepatic steatosis and the underlying molecular mechanisms. Male apolipoprotein-E knockout mice were fed with a high fat diet, and two groups received also flaxseed oil-treated (FO) or flaxseed oil combined with its ester of plant sterols-treated (FO-PS). The results showed a decrease of serum and liver triglycerides levels, serum levels of ALT and AST, reactive oxygen species (ROS) production, in mice fortified with FO-PS compared with the significantly higher values exhibited in HFD-fed mice only. Furthermore, the plasma levels of IL-6, TNF-α, MCP-1, and sICAM-1 were significantly lower in combined treatment than in high-fat diet. Moreover, an up-regulation of the genes expression of hepatic proliferator activated receptor alpha (PPARα) and genes involved in cholesterol efflux (ABCA1, LXR, SR-BI) was observed [40].

In 2017, Song et al. suggested that PSs would play an important role in reducing the evolution of NAFLD, already knowing the effects in lowering cholesterol, blood sugar, and antioxidants serum levels [41]. In his study, four-week-old male Sprague Dawley rats received a hyperlipidemic diet daily; the administered milk was enriched with different doses of phytosterol esters (PSE). During the study, hepato-renal function, lipid profile, histopathological liver changes, and the impact on proinflammatory cytokines, oxidative markers, and the gene related to lipid metabolism and inflammation were evaluated. The results of the study showed that PSE has changed favorably liver transaminases and lipid profile, correlated with improved histopathological status in rats with NAFLD. The human-rat equivalent of low and medium doses of PSE has been shown to have a protective effect on the onset and progression of NAFLD, by decreasing oxidative stress, serum LDL-C, and by modulating the cytokines involved. Thus, the need for additional studies in humans to confirm the therapeutic role of PSE in this pathology was emphasized [41].

Another aspect of NAFLD’s development is the association with intestinal dysbiosis. Song et al. investigated the impact of phytosterol esters (PSEs) on the intestinal microbiota by comparing the administration of high and low doses in adult Sprague Dawley rats. Those rats that received high doses of phytosterols in daily administration had a richer intestinal flora in *Bacteroidetes* and *Anaerostipes*. The authors argue that the effect on the intestinal microbiota and fecal metabolites is beneficial. The findings of this study opens up new opportunities for prevention and therapy in NAFLD [42].

Knowing that different forms of PSs have different implications in improving the lipid profile, Simin Feng et al. compared the effects of a pure stigmasterol and β-sitosterol dietary combination in experimental animals that developed NAFLD after receiving a high-fat Western-style diet (HFWD) [43]. The doses of phytosterols used were in accordance with those recommended by the FDA for human use. The authors hypothesized that PSs influence lipid absorption and metabolism by altering the reabsorption of cholesterol and acid bile. Total cholesterol, HDL-C, non-HDL-C, TG, and ALT levels were periodically dosed from serum; liver histological lesions were also assessed using a histological scoring system, as well as the degree of inflammation associated. Further evaluations were performed to assess the effects of genes involved in lipid and bile acid metabolism. The determination of lipidomics is an efficient and sensitive approach to the biological changes caused by lipid variations in the evaluation of NAFLD. The results showed that stigmasterol is more effective than β-sitosterol in reducing body weight gain, hepatic lipid accumulation, and alleviation of HFWD-induced NAFLD, secondary to decreasing the reabsorption or absorption of cholesterol, bile acids, and dietary lipids; these effects can be explained by altering the expression of some lipogenic genes and lowering circulating CM levels [43].

Another in vivo study published in the same year confirm the positive influence of stigmasterol and β-sitosterol on NAFLD induced by a high-fat Western diet in mice. The long-term (33 weeks) supplementation of 0.4% stigmasterol or β-sitosterol in the diet of mice included in the study had antilipidemic activity: reducing triacylglycerols, hepatic cholesterol, and disruption in liver-free fatty acids [44]. Most of these effects are the results of a decreased absorption of the lipid.

In 2020, Gumede et al. published two studies on the benefits of β-sitosterol in lipid metabolic disorders, as well as anti-obesogenic and antidiabetic effects. One study evaluated the protective potential of β-sitosterol in the onset and progression of NAFLD in growing rats fed with high levels of fructose; the authors correlated the developmental pattern in baby mice exposed to a diet unsuitable for physiological growth, with children exposed to obesogenic diets [45]. Markers of serum liver function were evaluated, which showed an increase in total liver lipids. At the end of the study, a macro and microscopic examination of the liver showed an increase in liver mass, the presence of areas of inflammation and lesions of micro- and macrovesicular hepatic steatosis. The groups of rats that received additional β-sitosterol/fenofibrate diet showed a favorable liver evolution compared to the groups with a high fructose diet, which reinforces the idea that β-sitosterol has a favorable effect in preventing/attenuating diet-induced NAFLD [45]. Knowing the favorable effects in ameliorating lipid metabolism disorders of PSs, Gumede et al. publishes another study on growing female rats, who received a diet rich in fructose; that type of diet is known as a risk factor for disorders of lipid metabolism and the development of metabolic syndrome, NAFLD, cardiovascular pathology. Basically, they mimicked the pediatric population that consumes a diet predisposing to obesity. Serum levels were assessed for the following parameters: glucose, insulin, cholesterol, triglycerides, adiponectin; the visceral fat was also weighed. The results obtained after the evaluation of the group that received a diet rich in fructose with supplementation of β-sitosterol, suggest that β-sitosterol could be used in the prevention of metabolic disorders induced by a diet rich in fructose. This favorable result is due to its proven effect in preventing hypertriglyceridemia, hypoadiponectemia, and visceral fat [46].

Endoplasmatic Reticulum (ER) stress, according to literature studies, represents a key factor in the onset and progression of NAFLD. The direct effect of ER stress is the activation of ER oxidoreductin 1, the consequence being the overproduction of Reactive Oxygen Species (ROS), followed by disorder in the β-oxidation of fatty acids, with intracellular storage for cholesterol and saturated fatty acids. In a study conducted by Hao Han et al. on laboratory animals receiving a high fat and high cholesterol diet, administration of plant sterol ester of α-linolenic acid (PS-ALA) decreased liver size and body mass index, significantly improved liver tissue degradation, reduced oxidative stress, inhibited Sterol Regulatory Element-Binding Protein (Srebp) pathway activation, ameliorated lipid metabolism and thus intracellular fat accumulation, as well as inflammatory reaction (IL-1β, IL-18) and hepatocytolysis. The authors concluded that supplementation with PS-ALA leads to improvement of β-oxidation of fatty acids, by inhibiting ROS overproduction and enhanced mitochondrial biogenesis, which means it plays an important role in improvement of NAFLD [47].

### 3.2. Clinical Trials

The promising results in animal models on the beneficial effects of PSs in NAFLD encouraged straying the role of PSs supplementation in human patients.

Cross-sectional studies have shown a correlation between decreased serum levels of plant sterols and NAFLD, suggesting that intrahepatic transport mechanisms may be affected. Tauriainen et al. studied the link between export mechanisms to the most common plant sterols and plant stanols present in humans and NAFLD [52]. The group included 138 obese individuals participating in the Kuopio Obesity Surgery Study (KOBS). Approximately one third of the participants did not have hepatic histological changes, the rest having characteristic features of NAFLD. Dietary phytosterol intake (DPI) was not different between the study groups. The following were evaluated: serum, hepatic, and bile plant sterols and stanols and liver mRNA expression of several genes regulating inflammation and lipid metabolism, as well as the evaluation of lipid metabolism in serum. The main idea was that liver sitosterol and sitostanol ratios to cholesterol vary in obese patients depending on the presence or absence of NAFLD, without correlation between serum levels of these biological compounds and histological lesions. The main observation was that in patients with NAFLD, hepatic and bile sitostanol levels were increased, showing a direct relationship with steatosis and lobular inflammation. For the future, it remains to be clarified by what mechanisms the disturbance of sitostanol metabolism intervenes in NAFLD [52].

In response to the aforementioned study, Plat et al. considers that there is a possibility that the results published in the study by Tauriainen et al. may be secondary to the applied analytical methodology. Thus, Plat et al. emphasized the need for placebo-controlled intervention studies to certify the effects of plant sitosterol and sitostanol on the liver, positive or negative [53]. They exemplify the study published by Javanmardi et al. [48] in which it was shown that the daily diet supplemented with phytosterols improved the biological profile of patients.

In 2018, Javanmardi et al. performed a double-blind clinical trial with 38 patients with NAFLD, organized into two randomized groups. One of the groups received 1.6 g of phytosterol daily. The results obtained showed that daily supplementation with phytosterols had a positive impact on LDL-C, TNF-α, ALT, and AST, strengthening the idea that phytosterols could be an effective therapy in NAFLD [48].

Another study of Chen et al., 2015, mentioned that administration of 1.8 g of PSs daily may improve insulin resistance, reduces LDLc concentrations, and downregulates systemic inflammation [49]. These two studies highlight the idea that more than 1.6 g/day of PSs may have a beneficial role in preventing or ameliorating the damage in NAFLD in adult patients.

A more recent study, based on the favorable results of phytosterol supplementation on lipid metabolism and the evolution of NAFLD, evaluated in a randomized, double-blind, placebo-controlled trial the effects of the administrations of PS (3.3 g/d, equal to 2.5 g phytosterol in the free form/d) plus n-3 PUFA (450 mg EPA + 1500 mg DHA) [50]. Anthropometric evaluations (body mass index, Waist circumference, Hip circumference), blood samples (Serum TC, TAG, HDL-cholesterol, LDL-cholesterol, glucose, liver and kidney function markers, TNF-α, TGF-β), and CT-scanning (for quantify the liver:spleen attenuation ratio) were performed at the beginning and end of the study. The study included NAFLD patients aged 30–67 years, with an average liver:spleen attenuation ratio of ≤1·2 or liver attenuation ≤52 Hounsfield units (HU), overweight or dyslipidemic. The findings were encouraging, showing the synergism of the mechanisms of action of PS + DHA + EPA in daily administration, more effective compared to separate or placebo administration, in reducing serum TAG, TC, and LDL-Cholesterol, decreasing the level of pro-inflammatory cytokines and improving liver damage. However, the authors emphasize the need for a large population trial with NAFLD subjects and simultaneous administration of phytosterol and n-3 Long-chain PUFA (especially EPA and DHA) [50].

Furthermore, the group of Song L et al. continued the study on the role of co-supplementation of n-3 PUFAs and PSE in metabolic disorders associated with NAFLD. After 12 weeks of co-supplementation of n-3 PUFAs and PSE in patients with NAFLD, they observed that serum levels of PUFA-containing phosphatidylcholine (PC), lysophosphatidylcholine (LysoPC), perillyl alcohol, and retinyl ester significantly increased. Therefore, metabolomic regulation may explain the potential role of co-supplementation of n-3 PUFAs and PSE in improving hepatic steatosis [54].

## 4. Conclusions and Perspectives

Due to the dramatic increase in NAFLD prevalence and the current lack of effective therapies, there is a great need to identify dietary approaches for NAFLD prevention and treatment. In this review we highlighted some recent studies into the beneficial modulatory effects of phytosterols for NAFLD intervention. The mechanisms underlying such observations are likely to include lipid metabolism regulation, to suppress the development of hepatic inflammation, modulate key cytokines involved in inflammation, decrease serum levels of ALT, AST, modulation of oxidative pathways, and in some situation improvement of the histopathological aspect of NAFLD. Moreover, current research on the circulating PSs indicates its safety concerns.

The encouraging results of these studies, both in terms of phytosterols administered as monotherapy of preventing the occurrence and slowing the NAFLD evolution, and in those administration in various combinations with other biologically active substances, represent a growing field of interest as an alternative to classic drug treatments. Experimental animal studies cannot predict the effects on the human population. In the coming years, long-term and large-scale clinical trials are needed to certify the preventive and therapeutic action of phytosterols in NAFLD.

Furthermore, studies are needed in the pediatric population, where some of the drug therapy used in adults is not yet approved for children; due to the peculiarities of age, we need safe and effective products with as few side effects and certain therapeutic results. Further clinical trials using dietary achievable doses are needed to support the use of phytosterols in NAFLD intervention, their bioavailability and safety issues.

## Data Availability

No new data were created or analyzed in this study. Data sharing is not applicable to this article.
